# Effect of Non-Meat Protein Addition on the 3D Printing Performance of Chicken Meat

**DOI:** 10.3390/foods14061015

**Published:** 2025-03-17

**Authors:** Xin Li, Mingyuan Huang, Dan Chen, Enquan Xiao, Yuqing Li

**Affiliations:** 1 College of Food and Bioengineering, Wuhu Institute of Technology, Wuhu 241003, China; lxin@whit.edu.cn (X.L.); yuqlio@163.com (Y.L.); 2 College of Food Engineering, Anhui Science and Technology University, Chuzhou 233100, China; 3 College of Food Science and Engineering, Yangzhou University, Yangzhou 225127, China; dchen@yzu.edu.cn; 4 College of Life Science, Anhui Normal University, Wuhu 241008, China; shawnxeq@163.com

**Keywords:** chicken meat, 3D printing, non-meat proteins, rheological properties, gel

## Abstract

In this study, three types of non-meat proteins, including soybean protein, wheat gluten, and whey protein, were used as additives to improve the 3D printing performance of chicken meat. The effects of non-meat proteins on rheological behavior, textural properties, moisture characteristics, and the microstructure of gels were investigated. Chicken meat paste without non-meat proteins added was taken as a control. Rheological results showed that the addition of non-meat proteins increased the apparent viscosity and the storage modulus of chicken meat paste. Textural properties of gels, including hardness, chewiness, cohesiveness, springiness, and resilience were also improved. The microstructure of gels with non-meat protein addition became denser and more compact, with improved connectivity. Nuclear magnetic resonance showed that the signals of bound water, immobilized water, and free water moved to the left towards lower relaxation time (*p* < 0.05) and part of immobile water and free water changed to bound water. The samples containing 15% soybean protein exhibited good shape-forming and shape-keeping capacities. There was an obvious increase in hardness (1991.40 ± 88.22 g), springiness (0.92 ± 0.00), cohesiveness (0.72 ± 0.01), gumminess (1299.14 ± 21.21), and resilience (0.34 ± 0.01) in these samples. The cooking loss of samples containing 15% soybean protein was 2.46 ± 0.36%, which was significantly lower than that of other treatments (*p* < 0.05). In summary, 15% soybean protein-added samples showed great potential for 3D printing.

## 1. Introduction

Chicken meat contains a high level of nutrients and is regarded as a cheap source of high-quality protein in human diets [1]. Globally, the consumption of chicken meat continues to rise and is progressively assuming a predominant role in the global meat market [2,3]. It is anticipated that by 2028, the global chicken market will expand by an astonishing amount of USD 429.11 billion [4]. In recent years, consumers have exhibited a higher demand for food that is capable of providing both rich nutrition and an appealing appearance. Consequently, the processing technology of chicken production demands innovation.

Three-dimensional printing is a kind of innovative food processing technology. By utilizing 3D printing, it is possible to blend specific materials and then print them into various geometric shapes that are unattainable or uneconomical to produce by conventional manufacturing methods [5]. In addition, 3D printing has the potential to personalize food products for people who require special nutrition, such as children, the elderly, athletes, or obese individuals [6]. A quite amount of research has been conducted on 3D printing for food, with a focus on the printability of various food ingredients [7]. Meat serves as a crucial source of nutrition for human consumption, and its printability has attracted considerable attention from numerous researchers. In 2010, the work of 3D printing meat was first reported by Lipton et al., who successfully printed turkey meat products, demonstrating the printability of meat for 3D printing [8]. In later research, many 3D printing products based on chicken, fish surimi, beef, and pork have been reported [9,10,11].

However, challenges to the application of 3D printing in meat still exist. During 3D printing, the meat paste can be regarded as a complex polyphase colloidal system. Water is considered as a continuous phase, while protein, fat, and inorganic salt as the dispersed phase. The myogenic fibers, as long-chain molecules, are fully hydrated in solution, leading to extrusion interruption during 3D printing [12]. The proper fluidity and viscosity of meat paste are crucial for smooth and accurate 3D printing [11], and better gel-forming ability of the matrix can enhance the shape stability of final products [13]. In previous studies, adjusting formulations was regarded as a feasible way to improve the printability of meat, such as lipids, transglutaminase, and hydrophilic colloids. Wang et al.’s proposed amount of NaCl addition had a significant impact on the rheological properties of meat, and gels made with a 1.5% NaCl (*w*/*w*) mixture were the most suitable for extrusion from a nozzle during 3D printing [14]. Dong et al. utilized sweet potato starch (8%, *w*/*w*) as a structural modifier to achieve stable 3D-printed surimi constructs [15]. Yu et al. added hydrocolloids into the meat paste, resulting in enhanced water retention capacity of gels and improved mechanical strength of 3D printed samples [16]. Transglutaminase was also used to promote cross-linking among proteins to improve the shape accuracy of surimi 3D products [17]. There has been relatively limited research on the 3D printing of chicken meat. Bulut et al. added gelatin into chicken meat and found that the addition of 1.79% gelatin is beneficial for enhancing printing performance [18]. However, the printing quality of chicken meat still needs to be elevated. Although there are numerous studies endeavored to optimizing formulations of3D printing meat, the information on non-meat proteins used as additives in 3D printing meat is very limited. In recent years, considering environmental, ethical, and health concerns, the production of non-animal proteins has garnered extensive attention from scholars [19,20]. There has been a growing interest in the intake of non-meat proteins in the human diet [21]. Non-meat proteins from different sources for 3D printing were also noticed by researchers [22,23,24]. Soybean protein, rich in essential amino acids and with low cost, has good processing properties, such as gel properties and water holding capacity [25]. Consequently, it has been used in meat processing to enhance product quality [26,27]. The printability of soybean protein has been approved. Chen et al. successfully used soybean protein as the main printing material at a content level of 79.5% to print steak-like food products with texture properties similar to chicken breast, which indicated that soybean protein had the potential to support the shapes of printing products [28]. Qiu et al. used soy protein- and wheat gluten-based pastes to construct 3D-printed meat analogs, and approved the printing capability of plant-based proteins [29]. Wheat gluten is a kind of economical protein resource, with excellent viscoelasticity, elongation, and thermosetting properties [30]. It has been used for 3D printing of dough [31] and plant-based meats [32]. Whey protein is a kind of protein extracted from milk, which is rich in nutrients and has the capacity to form co-gels with other food ingredients [33]. Du et al. confirmed that adding whey proteins at the level of 20% (*w*/*w*) could significantly enhance the 3D printing performance of konjac hybrid gels, in which the particles were more uniformly dispersed [34]. Therefore, there are many advantages, such as good processing properties, rich nutrition, ease of utilization, sustainability, healthfulness, and economic feasibility [35,36]. Nevertheless, the effect of non-meat protein addition on the quality improvement in 3D printing meat products is poor [12,37].

Based on the above, three different sources of proteins were chosen and added to chicken meat paste, including soybean protein, wheat gluten, and whey protein, which were essential for humans’ daily diet. The rheological behavior and 3D printing qualities of these mixed materials were evaluated. The aim of this study was to investigate the effect of non-meat protein addition on chicken meat 3D printing performance and to develop a printable formulation primarily consisting of non-meat proteins and chicken meat.

## 2. Materials and Methods

### 2.1. Materials

Fresh chicken breast and edible salt (NaCl, purity > 99%) were purchased from a local market in Wuhu, China. Soybean protein, wheat gluten (WG), and whey protein (WP) were purchased from Macklin Biochemical Technology Co., Ltd. (Shanghai, China).

The visible connective tissue and fat were trimmed from the meat and then minced using a mechanical mincer (JR18G-300, SUPOR, Hangzhou, China). In total, 1.5% NaCl (*w*/*w*) and a suitable quantity of chilled water were mixed in and blended for 3 min until the final moisture content reached 80% using a blender (SDD2001, SUPOR, Hangzhou, China), and chicken meat paste (CMP) was obtained, which was taken as control. Based on CMP, SP, WG, and WP were, respectively, added to CMP at three content levels as Table 1, and blended for another 3 min.

### 2.2. Rheology Measurement

The rheological measurement was conducted following a previous study by a dynamic rheometer (MCR 302e, Anton Paar, Graz, Austria) at 25 °C [38]. The samples were placed between a 25 mm parallel steel plate geometry and a platform, with a gap of 1 mm. The apparent viscosity was determined as the shear rate was ranging from 0.1 to 100 s^−1^. Storage modulus (G′) and loss modulus (G′′) were determined at angular frequency sweeping from 0.1 to 100 rad/s.

### 2.3. Printing Process

A solid cylinder (r = 10 mm, h = 20 mm) model was selected and printed by a 3D printer (S2PRO, FoodBot, Hangzhou, China). The printed samples were used in the determination of textural properties, cooking loss, low-field nuclear magnetic resonance, and microstructure. Also, a model of cuboid (25.00 mm × 25.00 mm × 5.00 mm) was constructed to demonstrate the printing performance of compound materials. Parameters of the 3D printing process were set as follows: 1.55 mm nozzle diameter, 2.0 mm nozzle height, 20 mm/s nozzle moving speed, and 0.002 cm^3^/s extrusion rate.

### 2.4. Texture Profile Analysis (TPA)

Texture measurement was evaluated by a texture analyzer (TA-XT Plus, Stable Micro Systems, London, Britain) according to a previous method with some modifications [16]. The samples were compressed at a deformation rate of 1 mm/s, and the pre-test and post-test rate was 5 mm/s. Each gel was compressed by a 50 mm diameter flat probe (P/50) with two consecutive compressions of 40%.

### 2.5. Cooking Loss

The samples were cooked at 100 °C for 10 min in a water bath (HH-4, LICHEN, Shanghai, China). After cooking, the surface water of the samples was moved and the cooking loss (%) was expressed as the weight of the lost water as a percentage of the weight before cooking [10].

### 2.6. Low-Field Nuclear Magnetic Resonance (LF-NMR)

The water distribution and migration within the samples were measured by an NMR spectrometer (PQ001, Niumag, Shanghai, China) according to a previous method with slight modifications [39]. About 2 g sample was weighed and put into an NMR tube (diameter of 15 mm). The test conditions were as follows: SW = 100 kHz, TW = 4000 ms, TE = 0.35, NECH = 7000, NS = 16. The Carr–Purcell–Meiboom–Gill (CPMG) sequences were used for transverse relaxation time (T_2_) measurement.

### 2.7. Scanning Electron Microscopy (SEM)

SEM measurement was conducted in accordance with a previous method with slight modifications [17]. The samples were placed in 2.5% (*v*/*v*) glutaraldehyde and stored for 2 h at 25 °C, followed by dehydration using different concentrations of ethanol. After being dried, the samples were sprayed with gold. The microstructure of the samples was observed by a scanning electron microscope (SU8100, HITACHI, Hitachi, Japan) operating at an accelerating voltage of 20 kV and a magnification of 1500 times.

### 2.8. Statistical Analysis

All the results were reported as mean ± standard deviation based on three independent determinations. Data analysis for one-way ANOVA and Duncan tests was conducted using the SPSS software (version 22.0, IBM, New York, NY, USA) at a confidence level of 95%.

## 3. Results

### 3.1. Rheological Properties

**Figure 1:**
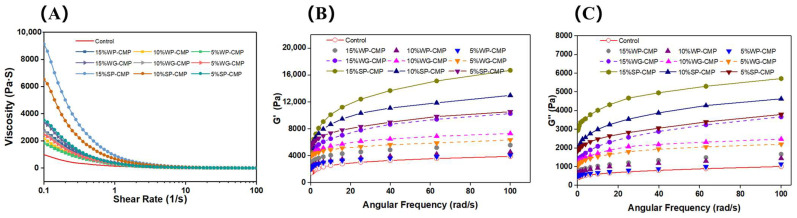
Dynamic rheological characteristics of chicken meat paste and samples with non-meat. protein addition ((**A**): apparent viscosity; (**B**): G′; (**C**): G′′).

The rheological properties of materials are crucial for extrusion-based 3D printing, which are closely related to their printing performance [40]. The apparent viscosity curves of the samples are displayed in Figure 1A. Irrespective of protein types, the viscosity decreased with the increased shear rate, suggesting that all the samples in the test were pseudoplastic fluids and showed shear-thinning behavior. For extrusion-based 3D printing, shear-thinning behavior enabled the materials to be easily extruded from nozzle [41]. When non-meat proteins were added to meat paste, the viscosity tended to increase, and soybean protein-added samples showed significantly higher viscosity than the other samples, and 15% SP-CMP had the maximum viscosity. These results could be explained that pores of the myofibrillar proteins gel network were filled by non-meat proteins, leading to the reduction in the fluidity of materials [24]. Soybean protein and wheat gluten have better water-holding capacity [26,30] and contribute to the retention of a large number of water molecules within the matrix, which leads to higher viscoelasticity of SP-CMP and WG-CMP.

The storage modulus (G′) and loss modulus (G′′) were measured to reflect the viscoelastic properties of the materials. The G′ reflects the elastic solid-like behavior and predicts the mechanical strength, while the G″ is the index reflecting the viscosity of materials [10]. The results of this study are depicted in Figure 1B,C. Among all the samples, G′ and G′′ both progressively increased with the growth in angular frequency. G′ was always much higher than G′′ in each sample, which reflecting the materials were in an elastic dominant state [42]. The addition of non-meat proteins enhanced the G′ values of meat paste. We found that rheological properties were significantly affected by the type of protein additives. The 5% SP-CMP, 10% SP-CMP, 15% SP-CMP, and 15% WG-CMP showed higher apparent viscosity and storage modulus than the other samples, which could lead to better printing behavior. However, the whey protein added samples showed lower G′, which could result in poor self-supporting capacity after printing. We speculated that the differences in rheological properties in these treatments are probably related to the different gel-forming abilities of proteins from different sources. Whey proteins are smaller globular milk proteins extracted from milk [33], and these protein molecules tend to aggregate granularly during the formation of gel [43]. When whey proteins are added to chicken meat, these granular aggregates fill in the gel matrix but lack a three-dimensional network structure. Conversely, soybean protein and wheat gluten have good viscoelasticity and water-holding capacity [25,30], which could promote the gel network formation in mixed gels.

Overall, the addition of non-meat proteins was helpful for reducing the fluidity and improving the elasticity of CMP. It was crucial for 3D printing improvement to choose the proper type of non-meat proteins and additive amounts.

### 3.2. Texture Profile Analysis

The texture properties of gels were measured, and hardness, springiness, cohesiveness, chewiness, and resilience were recorded as shown in Table 2. Hardness reflects the self-supporting and shape-keeping capacities of printing products [12]. The springiness and resilience are related to the ability to resist deformation during the 3D printing process [12]. It could be seen that the texture profiles were significantly affected by the type of protein additives. Soybean protein and gluten protein exhibited an obviously positive effect on meat gel texture; especially, 15% SP-CMP showed superior gel quality with maximum hardness, springiness, cohesiveness, chewiness, and resilience. Noticeably, 15% SP-CMP, 10% SP-CMP, and 15% WG-CMP exhibited higher springiness and resilience (*p* < 0.05) than the other treatments, suggesting that the samples tended to recover their original shape rapidly after being subjected to external force [24]. However, the hardness, springiness, gumminess, and resilience of 5% WP-CMP did not show a significant difference compared with the control. Furthermore, it was found that the cohesiveness values of whey protein added samples were lower than the control, which implied that the printing products would be easy to collapse [44].

These results demonstrated that the use of non-meat proteins as additives could enhance the textural characteristics of 3D-printed products. Soybean protein and wheat gluten were more expected than whey protein, especially treatments of 15% SP-CMP, 10% SP-CMP, and 15% WG-CMP. We speculated that non-meat protein filled in pores of gels and promoted cross-linking between proteins, thereby forming a uniform and compact gel network.

### 3.3. Cooking Loss of Gels

**Figure 2:**
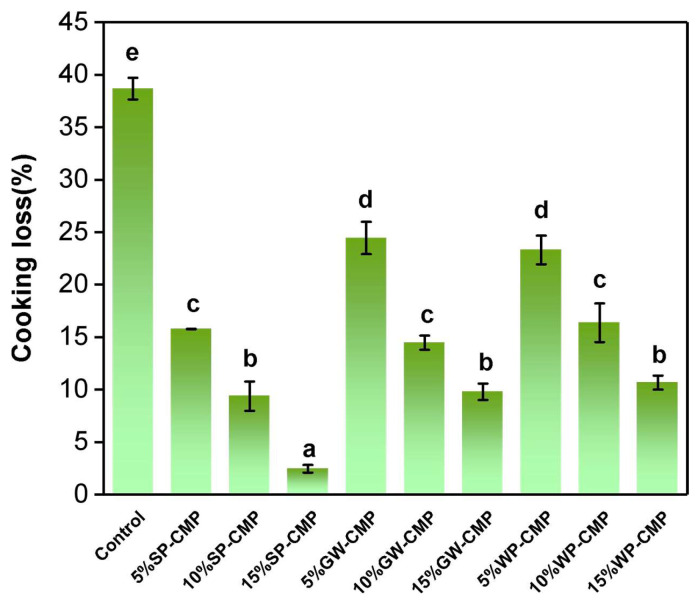
Cooking loss of chicken meat paste and samples with non-meat protein addition. Note: Means ± STD, *n* = 3. a to e, different lowercase letters indicated significant differences in the means.

Cooking loss was measured to evaluate the water-holding capacity of the samples, which has a critical influence on product yield and, in turn, has economic implications [45]. As shown in Figure 2, the cooking loss of non-meat proteins added gels was significantly lower than control. For the same addition level, the samples treated by the soybean protein displayed higher water-holding capacity than the samples treated by gluten or whey protein. Previous research proposed that gels with superior water-holding capacity typically exhibit high elasticity and hardness, and the internal water is not prone to loss, which indirectly reflects the stability of the microstructure of gels [6]. It is in accordance with the results of textural properties (shown in Table 2), which found that SP-CMP showed better gel texture. Furthermore, the cooking loss decreased with the non-meat protein content growing and the treatment of 15% SP-CMP showed the lowest cooking loss (*p* < 0.05). This result suggests that 3D-printed chicken products are feasible for steaming and cooking. And, 15% SP-CMP trend to show a high product yield, which is beneficial for enhancing the economic benefits of the product.

### 3.4. LF-NMR Analysis

**Figure 3:**
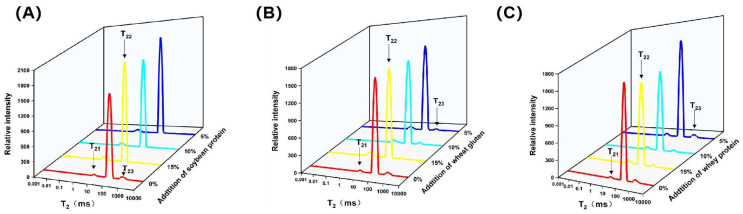
LF-NMR signal (T_2_) of chicken meat paste and samples with soybean protein (**A**), wheat gluten (**B**) and whey protein (**C**) addition.

The distributions and migration of different types of water in samples were determined by LF-NMR, and the results were displayed in Figure 3 and Table 3. Three relaxation times of samples approximately located in 1~10 ms (T_21_), 10~100 ms (T_22_), and 100~1000 ms (T_23_), which were regarded as the signals of bound water, immobilized water, and free water, respectively [46]. Compared to the control, T_21_, T_22_, and T_23_ all left-shifted towards the lower relaxation time (*p* < 0.05). In addition, the peak area proportion of immobile water (P_22_) and free water (P_23_) decreased and the proportion of bound water increased. These results indicated that the non-meat proteins limited the mobility of free and immobilized water, possibly by strengthening the binding ability of water molecules to gel network structure. As protein addition increased, T_22_ moved toward the left significantly. And, for the same addition level, meat paste with soybean protein addition exhibited lower relaxation time of immobilized water, reflecting better water-holding ability of these groups. The 15% SP-CMP showed the lowest T_22_ and the least free water, indicating that 15% SP-CMP had better water-holding ability than the other treatments, which was consistent with the findings of cooking loss. The impact of the non-meat proteins on water distribution and migration might be attributed to two reasons: one is the original hydrophilicity of various proteins, and that hydrophilic groups increased when non-meat proteins were filled in the gel matrix. Another was that a more denser gel network formed and thus more water was retained in the gel.

### 3.5. Microstructure of Gels

**Figure 4:**
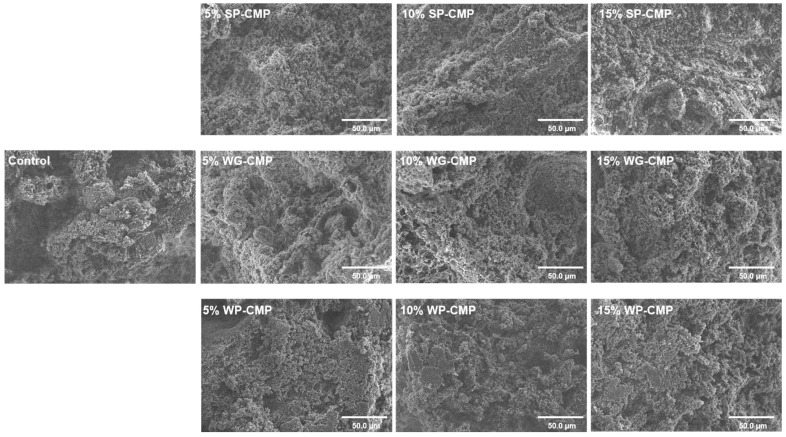
Micrographs of chicken meat paste and samples with non-meat protein addition.

The microstructure of the samples was observed by SEM and was exhibited in Figure 4. The results showed that significant differences in structure were observed between the control and the treated samples. Numerous and large holes formed in control, which presumably led printed products to collapse. During 3D printing, when the meat material passes through the nozzle tip, the bonds between the particles are broken under the shear force, making the structure of gels prone to be inhomogeneous [47]. Converse to the control, the non-meat protein addition treatments exhibited more smooth and compact structures, and the number and size of pores decreased with the growth of non-meat protein content in the mixed systems. In all the treatments, 15% SP-CMP, 10% SP-CMP, and 15% WG-CMP exhibited a more continuous and homogeneous structure. The results of microstructure revealed that non-meat proteins mainly acted as filling and gelling roles, which dissolved and dispersed into small molecules and interacted with meat proteins, further enhancing the cross-linking, reducing the empty spaces, and causing the gel structure to be more ordered.

### 3.6. Three-Dimensional Printing Performance

**Figure 5:**
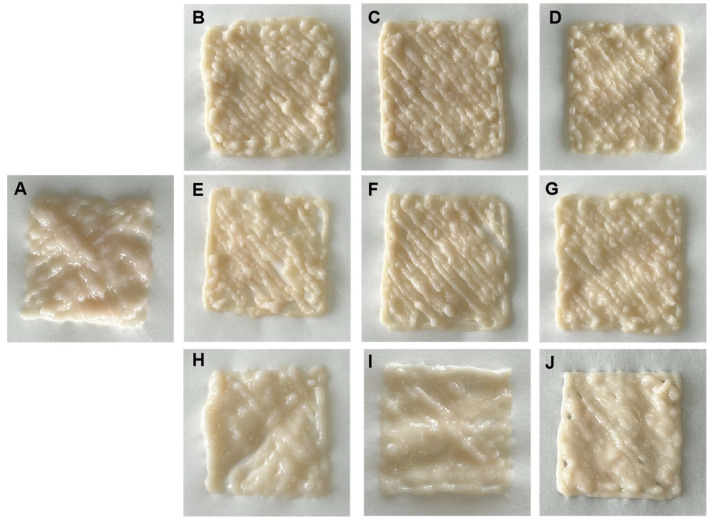
A demonstration of 3D printing samples of chicken meat paste and samples with non-meat protein addition. (**A**): control; (**B**): 5% SP-CMP; (**C**): 10% SP-CMP; (**D**): 15% SP-CMP; (**E**): 5% WG-CMP; (**F**): 10% WG-CMP; (**G**): 15% WG-CMP; (**H**): 5% WP-CMP; (**I**): 10% WP-CMP; (**J**): 15% WP-CMP.

The 3D printing performance of the samples is shown in Figure 5. The control exhibited inconsistent deposition lines and an undesirable appearance. Previous studies also reported that meat did not have the original 3D printing ability, caused by fiber structure and low viscosity [12]. Non-meat protein addition could improve the printability of chicken meat paste. For the soybean protein-added samples, the materials could be squeezed smoothly and maintain the stability of the spatial structures after deposition, which might be related to higher G′ in the samples illustrated in Figure 1B. The 15% SP-CMP showed superior printing behavior, with smooth extrusion and accurate appearance. It indicated that the whey protein-treated samples exhibited poor printing performance, with filaments merged and collapsed. It might be caused by the lower cohesiveness and resilience in these samples, which was demonstrated in our texture measurement (shown in Table 2). The printing behavior in the gluten addition treatments was inferior to the soybean protein addition treatments but better than the whey protein addition treatments. Broken lines could be observed in the 5% WG-CMP and 10% WG-CMP treatments, as gluten content increased, 15% WG-CMP showed an integral printing shape. Although apparent viscosity was improved in the gluten-added samples, the elasticity might be not enough to support the three-dimensional gel structure. Thus, the slight dragged and interrupted filaments were observed in the picture.

## 4. Conclusions

In this paper, the effect of non-meat proteins on the 3D printing performance of chicken meat was characterized. The results demonstrated that adding non-meat proteins could improve the apparent viscosity and storage modulus of materials, leading to enhancement of the 3D printing performance. Compared with whey protein, soybean protein and gluten had more positive effects on gel systems, including rheological behavior, textural properties, water distribution, and microstructure. Printing behavior gradually enhanced with the non-meat protein content growing. Combined with printing behavior, 15% SP-CMP was the optimal treatment, causing desirable shape-forming and shape-keeping abilities during the 3D printing process.

## Figures and Tables

**Table 1:** Chicken meat paste and non-meat proteins added meat paste formulations.

Ingredients (g)	Control	5%SP-CMP	10%SP-CMP	15%SP-CMP	5%WG-CMP	10%WG-CMP	15%WG-CMP	5%WP-CMP	10%WP-CMP	15%WP-CMP
CMP	100	100	100	100	100	100	100	100	100	100
SP	0	5	10	15	0	0	0	0	0	0
WG	0	0	0	0	5	10	15	0	0	0
WP	0	0	0	0	0	0	0	5	10	15

Note: The treatments with SP/CMP at a ratio of 5 g/100 g (*w*/*w*), 10 g/100 g (*w*/*w*), and 15 g/100 g (*w*/*w*) are named 5% SP-CMP, 10% SP-CMP, and 15% SP-CMP, respectively. The treatments with WG/CMP at a ratio of 5 g/100 g (*w*/*w*), 10 g/100 g (*w*/*w*), and 15 g/100 g (*w*/*w*) are named 5% WG-CMP, 10% WG-CMP, and 15% WG-CMP, respectively. The treatment with WP/CMP at a ratio of 5 g/100 g (*w*/*w*), 10 g/100 g (*w*/*w*), and 15 g/100 g (*w*/*w*) are named 5% WP-CMP, 10% WP-CMP, and 15% WP-CMP, respectively.

**Table 2:** Texture characteristics of chicken meat paste and samples with non-meat protein addition.

	Hardness (g)	Springiness	Cohesiveness	Gumminess	Resilience
control	826.02 ± 61.91 ^a^	0.811 ± 0.002 ^a^	0.69 ± 0.01 ^b^	568.95 ± 43.85 ^a^	0.27 ± 0.01 ^a^
5% SP-CMP	1097.69 ± 62.58 ^c^	0.87 ± 0.01 ^d^	0.72 ± 0.01 ^d^	715.46 ± 64.97 ^d^	0.30 ± 0.01 ^cd^
10% SP-CMP	1238.22 ± 94.22 ^de^	0.90 ± 0.01 ^e^	0.71 ± 0.02 ^cd^	826.80 ± 35.47 ^e^	0.342 ± 0.001 ^e^
15% SP-CMP	1991.40 ± 88.22 ^g^	0.922 ± 0.002 ^f^	0.72 ± 0.01 ^d^	1299.14 ± 21.21 ^g^	0.34 ± 0.01 ^e^
5% WG-CMP	1050.68 ± 58.11 ^c^	0.84 ± 0.01 ^c^	0.692 ± 0.004 ^bc^	672.06 ± 60.00 ^cd^	0.28 ± 0.01 ^ab^
10% WG-CMP	1177.88 ± 72.30 ^cd^	0.87 ± 0.01 ^d^	0.70 ± 0.01 ^bcd^	836.08 ± 56.59 ^e^	0.29 ± 0.01 ^bc^
15% WG-CMP	1418.08 ± 55.19 ^f^	0.892 ± 0.001 ^e^	0.72 ± 0.01 ^d^	1019.80 ± 48.84 ^f^	0.34 ± 0.01 ^e^
5% WP-CMP	837.78 ± 13.58 ^a^	0.82 ± 0.01 ^ab^	0.62 ± 0.01 ^a^	523.59 ± 10.34 ^a^	0.272 ± 0.001 ^a^
10% WP-CMP	944.10 ± 32.98 ^b^	0.83 ± 0.01 ^bc^	0.63 ± 0.01 ^a^	610.12 ± 57.85 ^bc^	0.27 ± 0.01 ^a^
15% WP-CMP	1062.48 ± 38.35 ^c^	0.84 ± 0.01 ^c^	0.63 ± 0.01 ^a^	738.11 ± 62.40 ^d^	0.28 ± 0.01 ^ab^

Note: Means ± STD, *n* = 3. a to g, different lowercase letters indicated significant differences in the means.

**Table 3:** T_2_ relaxation time and peak area proportion of the three populations of chicken meat paste and samples with non-meat protein addition.

	T_21_ (ms)	T_22_ (ms)	T_23_ (ms)	P_21_ (%)	P_22_ (%)	P_23_ (%)
Control	7.75 ± 0.32 ^f^	71.49 ± 0.00 ^g^	489.49 ± 17.24 ^f^	1.09 ± 0.06 ^a^	96.23 ± 0.22 ^de^	2.68 ± 0.24 ^g^
5%SP-CMP	1.82 ± 0.08 ^a^	56.07 ± 0.00 ^e^	460.59 ± 0.00 ^e^	3.69 ± 0.06 ^f^	95.86 ± 0.01 ^cd^	0.45 ± 0.05 ^b^
10%SP-CMP	1.63 ± 0.08 ^a^	45.21 ± 2.14 ^c^	381.27 ± 18.06 ^c^	3.30 ± 0.04 ^de^	96.53 ± 0.97 ^e^	0.12 ± 0.02 ^a^
15%SP-CMP	1.59 ± 0.00 ^a^	37.40 ± 0.00 ^a^	329.27 ± 7.00 ^b^	3.33 ± 0.05 ^de^	96.35 ± 0.43 ^e^	0.051 ± 0.003 ^a^
5%WG-CMP	5.81 ± 0.12 ^e^	62.51 ± 2.96 ^f^	364.95 ± 6.44 ^c^	3.08 ± 0.11 ^bc^	95.47 ± 0.33 ^c^	1.26 ± 0.06 ^c^
10%WG-CMP	4.95 ± 0.40 ^bc^	53.16 ± 2.52 ^d^	335.75 ± 4.53 ^b^	3.24 ± 0.05 ^cd^	94.78 ± 0.08 ^b^	1.98 ± 0.10 ^e^
15%WG-CMP	4.68 ± 0.22 ^bc^	40.56 ± 0.00 ^b^	282.64 ± 1.15 ^a^	5.33 ± 0.08 ^g^	93.81 ± 0.45 ^a^	1.06 ± 0.24 ^c^
5%WP-CMP	5.82 ± 0.47 ^e^	60.80 ± 0.00 ^f^	521.59 ± 20.52 ^g^	2.95 ± 0.07 ^b^	94.79 ± 0.06 ^b^	2.25 ± 0.13 ^f^
10%WP-CMP	5.22 ± 0.24 ^cd^	51.71 ± 0.00 ^d^	405.04 ± 11.55 ^d^	4.20 ± 0.24 ^h^	93.92 ± 0.25 ^a^	1.88 ± 0.07 ^de^
15%WP-CMP	4.32 ± 0.20 ^b^	43.98 ± 0.00 ^c^	361.23 ± 0.00 ^c^	3.46 ± 0.19 ^e^	94.86 ± 0.17 ^b^	1.68 ± 0.09 ^d^

Note: Means ± STD, *n* = 3. a to h, different lowercase letters indicated significant differences in the means.

## Data Availability

The original contributions presented in the study are included in the article, further inquiries can be directed to the corresponding author.
